# Cardiovascular Toxicity of BTK Inhibitors: A Patient-Centered Framework for Risk Stratification and Management

**DOI:** 10.3390/jcm15166160

**Published:** 2026-08-08

**Authors:** Andrea Tedeschi, Federico Barocelli, Luigi Gerra, Federico Breviario, Francesco Sbarra, Gianluca Pagnoni, Giuseppe Marasacchia, Francesco Marangi, Susan Darroudi, Francesco Di Spigno, Francesca Coppi, Annalisa Arcari, Giulia Losi, Daniele Vallisa, Giampaolo Niccoli, Daniela Aschieri, Alessandro Navazio, Luigi Tarantini

**Affiliations:** 1 Cardiology Unit, Guglielmo da Saliceto Hospital, 29121 Piacenza, Italy; andrea.tedeschimd@gmail.com (A.T.); l.gerra@ausl.pc.it (L.G.); f.breviario@ausl.pc.it (F.B.); f.sbarra@ausl.pc.it (F.S.); g.pagnoni@ausl.pc.it (G.P.); f.dispigno@ausl.pc.it (F.D.S.); d.aschieri@ausl.pc.it (D.A.); 2 Cardiology Division, Parma University Hospital, 43126 Parma, Italy; federico.barocelli@gmail.com (F.B.); giampaolo.niccoli@unipr.it (G.N.); 3 Cardiology Division, Department of Biomedical Metabolic and Neural Sciences, University of Modena and Reggio Emilia, 41125 Modena, Italy; giuseppemasaracchia68@gmail.com (G.M.); francesco.marangi2410@gmail.com (F.M.); 4 Cardiology Unit, Department of Medical and Surgical Sciences for Children and Adults, University of Modena and Reggio Emilia, 41124 Modena, Italy; darroudis921@gmail.com (S.D.); francesca.coppi@unimore.it (F.C.); 5 Hematology Unit, Guglielmo da Saliceto Hospital, 29121 Piacenza, Italy; a.arcari@ausl.pc.it (A.A.); d.vallisa@ausl.pc.it (D.V.); 6 Clinical Hematology Department, Institut Català d’Oncologia-Hospitalet, 08908 Barcelona, Spain; giulia.losi09@gmail.com; 7 Cardiology Unit, Department of Specialized Medicine, AUSL-IRCCS in Tecnologie Avanzate e Modelli Assistenziali in Oncologia, 42100 Reggio Emilia, Italy; alessandro.navazio@ausl.re.it

**Keywords:** Bruton tyrosine kinase inhibitors, cardio-oncology, cardiovascular toxicity, chronic lymphocytic leukemia

## Abstract

Bruton tyrosine kinase inhibitors have transformed the management of chronic lymphocytic leukemia and other B-cell malignancies, but their clinical use is increasingly influenced by cardiovascular safety. In contemporary practice, patients receiving these therapies are typically older and characterized by a high burden of comorbidities, including cardiovascular disease, metabolic disorders, and renal impairment. In this setting, cardiovascular complications should not be interpreted as isolated adverse events, but rather as clinical expressions of underlying patient vulnerability and frailty. Atrial fibrillation and arterial hypertension represent the most frequent complications, while heart failure, ventricular arrhythmias, and bleeding, although less common, may carry significant prognostic implications. Importantly, these events often arise from the interaction between drug exposure and pre-existing comorbidities, and may lead to treatment interruption, dose reduction, or discontinuation, ultimately compromising the long-term benefit of otherwise highly effective therapies. This review provides a comprehensive overview of the clinical burden, mechanisms, and management of cardiovascular complications associated with these agents, and proposes a practical, patient-centered framework to guide clinical decision-making. A structured approach based on risk assessment, early detection, and proactive management is essential to prevent complications, optimize treatment continuity, and preserve the long-term benefit of therapy in a complex and vulnerable population.

## 1. Introduction

The therapeutic landscape of chronic lymphocytic leukemia (CLL) has been reshaped over the past decade by Bruton tyrosine kinase inhibitors (BTKis), now a cornerstone of disease management [1,2]. BTKis include covalent, irreversible agents—ibrutinib, acalabrutinib, and zanubrutinib—and the newer, non-covalent reversible inhibitor pirtobrutinib, developed to overcome resistance mechanisms such as BTK C481 mutations [2,3,4]; their clinical use extends beyond CLL to other B-cell malignancies, including mantle cell lymphoma, Waldenström macroglobulinemia, and marginal zone lymphoma, further broadening the relevance of their cardiovascular safety profile [4]. Despite these advances, treatment selection is increasingly driven not only by disease biology but also by patient-related factors, particularly comorbidities [5,6]. CLL and related B-cell malignancies predominantly affect older, multimorbid patients [5], in whom comorbidities substantially affect treatment tolerability—increasing the likelihood of adverse events, dose modifications, or discontinuation—and thereby shape real-world outcomes [6]. Within this framework, cardiovascular toxicity has emerged as a major determinant of BTKis use [7]. The first-in-class agent ibrutinib demonstrated remarkable efficacy but was also associated with a distinct spectrum of cardiovascular adverse events, including atrial fibrillation (AF), arterial hypertension (HTN), bleeding, and, less frequently, heart failure (HF) and ventricular arrhythmias (VAs) [8,9], largely related to off-target kinase inhibition and prone to accumulate with continuous administration. Although newer-generation BTKis have improved selectivity and a more favorable safety profile, cardiovascular risk remains clinically relevant and is not fully abrogated [10], and may compromise disease control indirectly, through dose reduction, treatment interruption, or discontinuation of otherwise effective therapy [11]. In this setting, cardiovascular toxicity should not be regarded as an isolated adverse event, but rather as a dynamic factor interacting with patient vulnerability, comorbidity burden, and therapeutic decision-making—a complexity only partially addressed in current cardio-oncology frameworks, including European Society of Cardiology (ESC) guidelines [12], where management often remains largely empirical. Understanding who the patient is—rather than simply which toxicity occurs—is therefore essential for interpreting the clinical relevance of adverse events and guiding treatment choices. Accordingly, this review aims to shift the focus from toxicity itself to the patient in whom it occurs, providing a practical, patient-centered framework to guide the management of BTKis therapy in a high-risk, comorbid population (Graphical Abstract).

## 2. Chronic Lymphocytic Leukemia in 2026: Therapeutic Strategies and Clinical Implications for Cardiologists

The advent of BTKis has redefined the therapeutic paradigm of CLL and other B-cell malignancies, shifting treatment toward continuous, targeted strategies capable of achieving sustained disease control. Mechanistically, BTKis inhibit Bruton tyrosine kinase within the B-cell receptor signaling pathway—covalently or reversibly, depending on the agent—thereby suppressing malignant B-cell proliferation and survival; off-target inhibition of structurally related kinases, particularly the TEC family (TEC, ITK, BMX, TXK), is thought to underlie much of their cardiovascular toxicity, as summarized in the Graphical Abstract [4]. Ibrutinib, the first-in-class agent, demonstrated a clear survival benefit over chemoimmunotherapy and rapidly became a standard of care across multiple treatment settings [13,14]. However, early clinical experience consistently revealed a non-negligible cardiovascular toxicity profile, including AF, HTN, bleeding, and, less frequently, HF and VA [7]. These observations have driven the development of more selective BTKis aimed at preserving efficacy while improving tolerability. Second-generation agents, such as acalabrutinib and zanubrutinib, have demonstrated comparable clinical efficacy with a consistently lower incidence of AF and a more favorable overall cardiovascular profile in randomized comparisons [14,15,16]. Although absolute differences in event rates may appear modest, their clinical relevance becomes substantial in the context of long-term exposure, where even incremental reductions in toxicity translate into improved treatment sustainability. More recently, non-covalent BTKis, including pirtobrutinib, have further expanded the therapeutic landscape, particularly in patients previously exposed to covalent inhibitors. Early data suggest a favorable safety profile, with a lower incidence of arrhythmic events, likely reflecting increased kinase selectivity. However, follow-up remains limited, and long-term cardiovascular risk cannot yet be considered fully characterized [17]. The main characteristics, current indications, and cardiovascular safety profiles of available BTKis are summarized in Table 1 [2,18,19,20,21,22,23,24]. Importantly, BTKis are typically administered as continuous therapies, often for prolonged periods, exposing patients to cumulative cardiovascular risk over time. In contrast, alternative strategies based on venetoclax—alone or in combination with anti-CD20 monoclonal antibodies—are generally time-limited, offering deep remissions with a predefined duration of treatment [14]. This distinction has direct clinical relevance, as it introduces different patterns of cardiovascular risk exposure that should be explicitly considered during therapeutic decision-making. Taken together, the evolution of BTKis therapy reflects a progressive refinement of the benefit–risk balance, in which cardiovascular safety has emerged as a key differentiating factor across drug generations. For both hematologists and cardiologists, this is not a secondary consideration: different therapeutic strategies expose patients to distinct trajectories of cardiovascular risk. As a result, treatment choice itself becomes a determinant of cardiovascular outcome, reinforcing the need for integrated, multidisciplinary management.

## 3. The Chronic Lymphocytic Leukemia Patient: Comorbidities as a Determinant of Outcomes

CLL predominantly affects older adults, with a median age at diagnosis exceeding 70 years, and the typical patient is therefore defined by multimorbidity and reduced physiological reserve as much as by disease biology [25]. Cardiovascular disease—particularly HTN and arrhythmias—together with diabetes and renal impairment constitute the most frequent comorbidities, generating a competing-risk scenario in which non–cancer-related mortality may rival leukemia-related mortality in older populations [26]. Comorbidity burden is a well-established, independent prognostic factor, and validated tools such as the Cumulative Illness Rating Scale (CIRS) and the CLL-specific comorbidity index (CLL-CI) allow structured risk stratification beyond disease-specific variables [27]. Beyond prognosis, a higher comorbidity burden reduces treatment feasibility, increasing the likelihood of adverse events, interruptions, and early discontinuation of continuous therapies such as BTKis [28]. Because chronological age alone is an inadequate surrogate for treatment tolerance, biological fitness, functional status, and frailty—captured through geriatric assessment—more accurately reflect patient vulnerability and increasingly inform routine evaluation [29]. Overall, the modern CLL patient should be regarded as a multimorbid individual in whom comorbidity burden shapes prognosis, treatment exposure, and outcomes, reinforcing the rationale for a patient-centered approach to BTKis management.

## 4. Cardiovascular Toxicity of BTK Inhibitors: Clinical Framework and Mechanistic Basis

Cardiovascular toxicities associated with BTKis should be interpreted within a structured and clinically oriented framework, as their relevance extends beyond isolated events to their impact on treatment continuity and overall patient outcomes [7,10]. In this context, cardiovascular events are not merely adverse effects, but dynamic components of the therapeutic pathway that may influence both drug exposure and disease control. To ensure a consistent and practical approach, each toxicity will be addressed according to key clinical questions: (1) incidence and clinical burden; (2) timing of onset; (3) identification of patients at increased risk; (4) diagnostic strategies, including the role of systematic and opportunistic screening; and (5) management and implications for oncologic treatment, including dose modification, temporary interruption, or therapeutic switching. Given the heterogeneity of available evidence, each section will be complemented by a summary table reporting the most relevant clinical studies across different BTKis, providing a concise and comparative overview of current data. For each toxicity, evidence from randomized controlled trials is systematically complemented by real-world registries, retrospective cohorts, and pharmacovigilance data, which are essential to capture the true incidence and clinical impact of cardiovascular events in routine, unselected practice.

### 4.1. Arterial Hypertension

**Figure 1:**
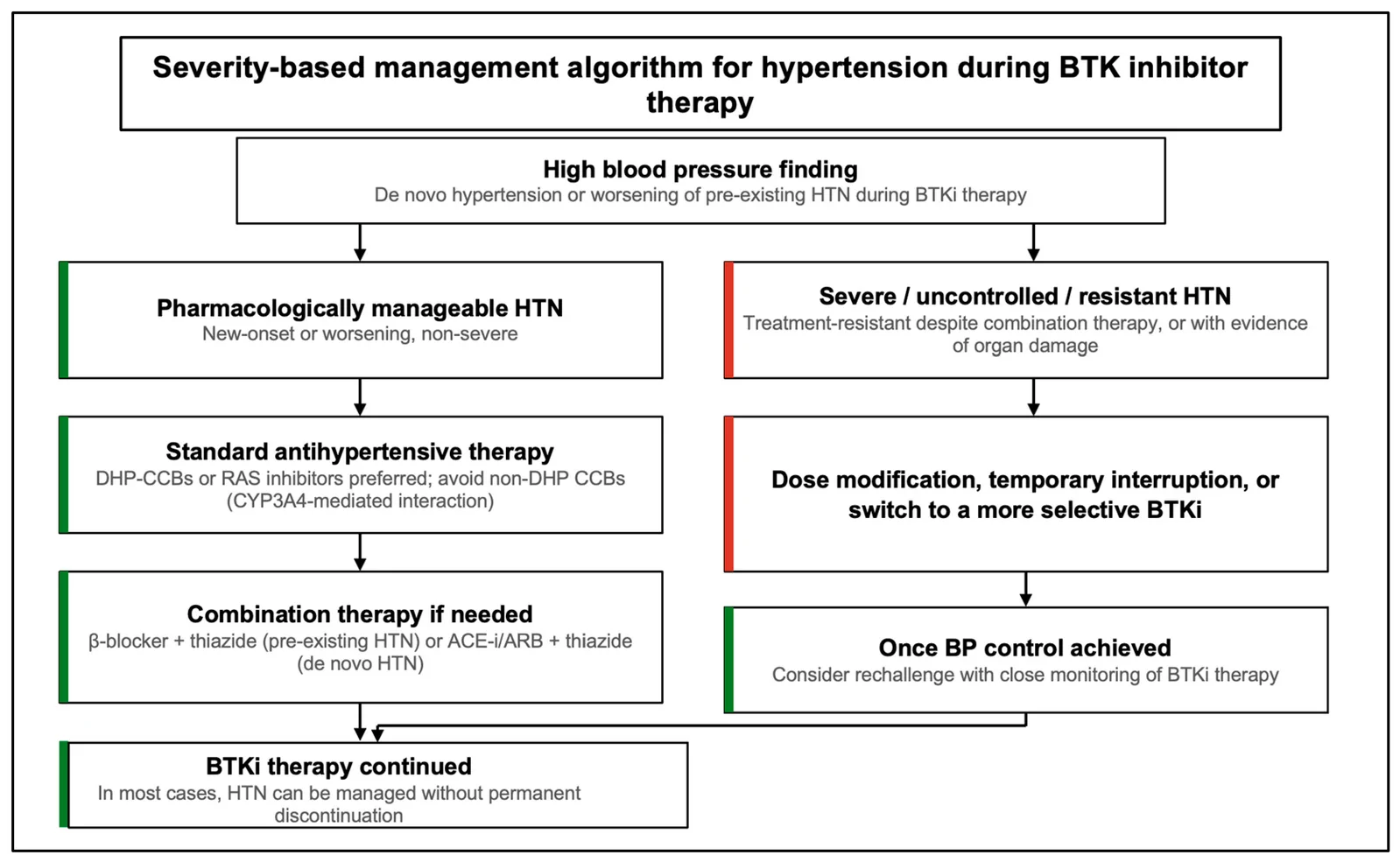
Severity-based management algorithm for hypertension during BTK inhibitor therapy: pharmacologic escalation, generation switch, and interruption criteria. Abbreviations: ACE, angiotensin converting enzyme; ARB, angiotensin receptor blocker; BP, blood pressure; BTKi, Bruton tyrosine kinase inhibitor; CCB, calcium channel blocker; CYP3A4, cytochrome P450 3A4; DHP, dihydropyridine; HTN, arterial hypertension.

HTN is among the most frequent and clinically relevant cardiovascular toxicities associated with BTKis therapy, particularly in the setting of long-term exposure [7]. Early clinical trials likely underestimated its incidence, whereas real-world and extended follow-up data have demonstrated substantially higher rates, especially with first-generation agents [30]. Reported incidence and patterns of BTKis-associated HTN across clinical trials and real-world studies are summarized in Table 2 [21,30,31,32,33,34,35,36]. With ibrutinib, grade ≥3 HTN has been reported in up to 25–30% of patients, while new-onset or worsening HTN may occur in a large proportion of treated individuals [31]. Although second-generation BTKis appear to be associated with a lower incidence, HTN remains clinically relevant, supporting at least a partial class effect [37]. This lower incidence with second-generation agents is directly supported by head-to-head randomized comparisons: in ELEVATE-RR, any-grade HTN occurred in 9.4% of acalabrutinib-treated patients versus 23.2% with ibrutinib [21], while in ASPEN, HTN incidence was 14.9% with zanubrutinib versus 25.5% with ibrutinib [36]. Indirect comparative analyses suggest a more favorable blood pressure profile with acalabrutinib compared with zanubrutinib in treatment-naïve patients [32]. From a temporal perspective, HTN may develop early after treatment initiation or emerge progressively over time [7]. While some patients experience blood pressure increases within the first months, others show a gradual rise during prolonged exposure. This is particularly relevant during continuous therapy, where uncontrolled blood pressure may drive cumulative vascular effects [26]. The underlying mechanisms of BTKi-associated HTN are not fully elucidated but are likely to involve endothelial dysfunction and altered vascular tone, potentially mediated by impaired nitric oxide signaling and off-target kinase inhibition [38]. The absence of a comparable hypertensive profile with other agents targeting overlapping pathways suggests a more complex and possibly drug-specific interplay. Patients at increased risk are typically older individuals with pre-existing cardiovascular disease, particularly those with baseline HTN [33]. Additional risk factors include diabetes, chronic kidney disease, and prior cardiovascular events. In these patients, the choice of BTKis may influence the overall cardiovascular risk profile, with more selective agents generally preferred when clinically appropriate. Diagnosis relies on systematic blood pressure monitoring, which should be implemented early and maintained throughout treatment. Home blood pressure monitoring represents a practical and effective strategy to detect early changes and guide treatment adjustments, particularly during the initial phases of therapy [15]. Management follows standard antihypertensive strategies, with careful attention to drug–drug interactions. Dihydropyridine calcium channel blockers and renin–angiotensin system inhibitors are generally preferred, whereas non-dihydropyridine calcium channel blockers should be avoided due to CYP3A4-mediated interactions [39]. Close monitoring is required, especially during the first months of therapy, and treatment intensification should be prompt in the presence of rising blood pressure. Combination therapy is often necessary. In a multicenter retrospective study, β-blockers plus thiazide diuretics were more effective in patients with pre-existing HTN, whereas angiotensin converting enzyme (ACE) inhibitors or angiotensin receptor blockers (ARB) combined with thiazides were more effective in patients with de novo HTN [34]. In most cases, HTN can be effectively managed without discontinuation of BTKis therapy. However, in patients with severe, uncontrolled, or treatment-resistant HTN, dose modification, temporary interruption, or treatment switching may be required. Importantly, the development of HTN is not a benign finding, as it has been associated with an increased risk of major cardiovascular events, including AF and HF [32] (Figure 1).

**Key points**
Frequent and often progressive toxicity, particularly with ibrutinib, with higher incidence in real-world and long-term exposure; associated with an increased risk of AF, HF, and major cardiovascular events.Risk stratification and early detection are essential, especially in older patients with baseline HTN or cardiovascular comorbidities; home blood pressure monitoring should be implemented early.Management is proactive and rarely requires discontinuation, but often necessitates combination antihypertensive therapy, close monitoring, and, in selected cases, dose modification or switching to more selective BTKis.

### 4.2. Heart Failure

HF is a less frequent cardiovascular toxicity than AF and HTN in patients treated with BTKis, and it has been predominantly reported with first-generation agents, particularly ibrutinib. Although uncommon, HF represents a clinically relevant complication, often occurring in association with other cardiovascular events [37]. Early clinical trials did not identify a clear HF signal; however, longer follow-up and real-world data suggest an incidence of approximately 2–5%, typically with late onset [37,40]. The true burden is likely underestimated, as most oncologic studies were not designed to systematically capture cardiovascular outcomes. Reported incidence and clinical patterns of BTKis-associated HF across clinical trials and real-world cohorts are summarized in Table 3 [40,41,42,43]. Real-world evidence supports an increased HF risk with ibrutinib. In a retrospective cohort of 860 patients with CLL, HF incidence was higher in patients receiving ibrutinib compared with chemotherapy (7.7% vs. 3.6% at nearly three years) [41]. Pharmacovigilance analyses further suggest a more than threefold increased reporting rate of HF with ibrutinib [42]. Taken together, these findings indicate a consistent, generation-dependent HF signal that is best documented with ibrutinib, whereas evidence for newer agents remains comparatively sparse. Data on newer BTKis remain limited. Acalabrutinib has shown a more favorable profile, with symptomatic HF reported in <1% of patients in pooled analyses, although long-term data are still scarce [43]. The absence of a clear HF signal with newer agents, including zanubrutinib, likely reflects limited systematic adjudication rather than a true absence of risk. HF may develop at variable time points, more often after prolonged exposure, and frequently in conjunction with other toxicities, particularly HTN and AF, suggesting a multifactorial pathophysiology. Patients at higher risk include those with pre-existing cardiovascular disease, especially prior HF, reduced left ventricular ejection fraction (LVEF), HTN, or AF, as well as older individuals [40]. Mechanistically, off-target inhibition of the PI3K–Akt pathway—critical for cardiomyocyte survival—may contribute to myocardial dysfunction, fibrosis, and impaired calcium handling. Additional contributors include endothelial dysfunction, altered nitric oxide signaling, and HTN-mediated remodeling, often leading to diastolic dysfunction [10]. Management requires prompt recognition and a multidisciplinary approach. Pre-existing HFrEF should be considered a relative, rather than absolute, contraindication to BTKis therapy, particularly with ibrutinib [44]. In higher-risk patients, second-generation BTKis are generally preferred, although they do not fully eliminate risk. In patients with new-onset or worsening symptomatic HF, treatment decisions should be guided by clinical severity. Initial management includes blood pressure control and volume optimization, followed by early initiation of GDMT, including beta-blockers, MRA, and SGLT2 inhibitors when indicated [45,46,47]. In selected cases with advanced disease or hemodynamic instability, escalation to more intensive therapies, including temporary inotropic support, may be required [48]. According to CTCAE criteria, grade 3 HF corresponds to symptomatic disease requiring hospitalization, whereas grade 4 reflects life-threatening conditions [49]. In severe cases (grade 3–4), treatment discontinuation and alternative oncologic strategies are generally recommended. In milder cases (grade 1–2), dose reduction or switching to a more selective BTKis may be considered [10]. From a prognostic perspective, HF during BTKis therapy is associated with increased cardiovascular morbidity, higher rates of treatment interruption, and worse overall survival [7]. Overall, the association between BTKis and HF is supported by consistent signals across real-world and pharmacovigilance data, but remains less robust than for AF and HTN due to the lack of systematic adjudication in clinical trials. This likely contributes to under-recognition and underscores the need for prospective cardiovascular-focused studies.

**Key points**
Less frequent but clinically significant toxicity, typically occurring with prolonged exposure and often in association with HTN and AF, reflecting a multifactorial pathophysiology.High-risk patients include those with pre-existing cardiac disease or reduced LVEF, in whom careful baseline assessment and closer monitoring are essential.Management often impacts cancer therapy, requiring early GDMT initiation, temporary interruption, or switching strategy; severe cases may necessitate permanent discontinuation.

### 4.3. Atrial Fibrillation

**Figure 2:**
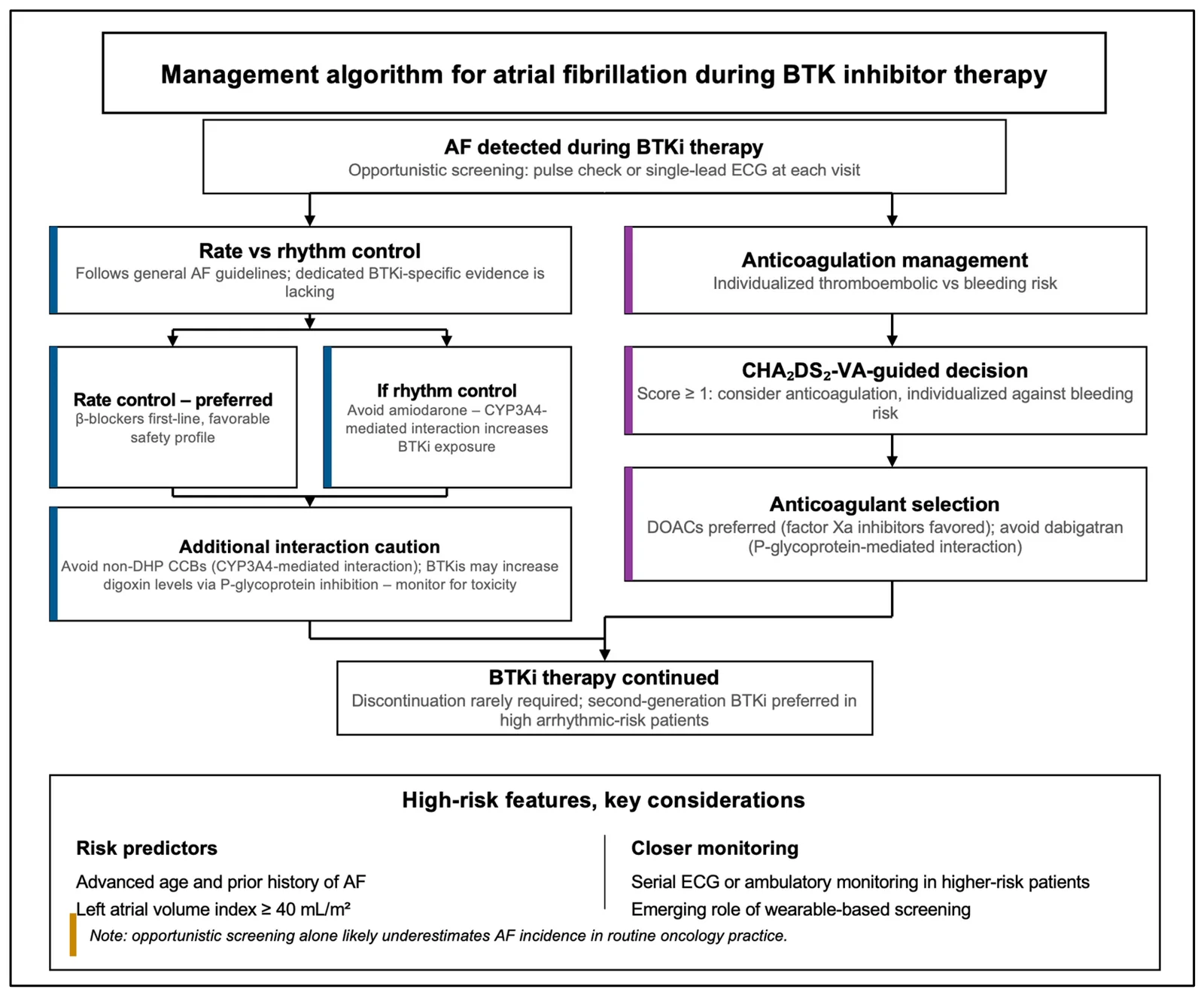
Management algorithm for atrial fibrillation during BTK inhibitor therapy: rate versus rhythm control, thromboembolic risk stratification, and anticoagulant selection. Abbreviations: AF, atrial fibrillation; BTKi, Bruton tyrosine kinase inhibitor; CCB, calcium channel blocker; CHA_2_DS_2_-VA, stroke risk score used for anticoagulation decision-making in atrial fibrillation; CYP3A4, cytochrome P450 3A4; DOAC, direct oral anticoagulant.

AF represents the most common cardiovascular toxicity associated with BTKis and a key determinant of treatment tolerability and long-term outcomes [7]. Initial oncology clinical trials reported new-onset AF in approximately 5–17% of patients, with severe symptomatic episodes occurring in up to 9% of cases [50]. Retrospective analyses suggest a median time to onset of 5 to 8 months, although AF may develop at any stage during therapy [51]. A meta-analysis of phase II–III trials reported the highest rates with ibrutinib, while other targeted therapies have also been associated with an increased AF risk, suggesting that this complication is not exclusive to first-generation BTKis [52]. Randomized trials consistently demonstrated a lower incidence with second-generation agents. In the ELEVATE-RR trial, AF occurred less frequently with acalabrutinib compared with ibrutinib (9% vs. 16%) [21], and similar findings were observed with zanubrutinib, with an overall relative reduction of approximately 40–45% in symptomatic AF events [36]. However, studies incorporating systematic cardiac monitoring indicate that AF is likely under-recognized in routine oncology settings. In prospective surveillance cohorts, cumulative AF incidence with ibrutinib approached 38% at two years, while extended ambulatory monitoring identified incident AF in up to 16% of patients, with a substantial proportion of asymptomatic episodes [53]. Consistent with these findings, large real-world analyses have reported a markedly increased long-term risk, with a 5-year AF incidence of approximately 25% in BTKis-treated patients and a more than fivefold higher risk compared with non-exposed populations [54]. Taken together, these data indicate that AF is a frequent and likely underestimated complication of BTKis therapy. The incidence, clinical context, and comparative risk across different BTKis are summarized in Table 4 [2,3,19,20,24,30,31,50,51,52,53,54,55,56,57,58,59,60,61]. The identification of patients at increased risk remains challenging. Advanced age and a prior history of AF have been consistently identified as the strongest predictors, while traditional cardiovascular risk factors, including HTN and HF, further contribute to susceptibility [55]. Furthermore, cardiac-focused studies have shown that structural remodeling of the left atrium, particularly atrial enlargement, is associated with a higher risk of AF [62]. Specifically, in a study of 54 patients treated with ibrutinib, a left atrial volume index ≥40 mL/m^2^ remained the only independent predictor of AF after multivariate analysis, highlighting the central role of left atrial remodeling and atrial cardiomyopathy [63]. The mechanisms underlying BTKis-associated AF remain incompletely understood. Proposed pathways include inhibition of C-terminal Src kinase, alterations in PI3K/AKT signaling, and modulation of cardiac ion channels, ultimately promoting atrial inflammation, fibrosis, and electrical remodeling [64]. Notably, the persistence of AF risk with more selective, next-generation BTKis suggests that additional or partially overlapping mechanisms may be involved [65,66]. From a clinical perspective, arrhythmia monitoring in oncology trials has been heterogeneous and largely limited to single ECG recordings during routine visits, likely contributing to underestimation of AF incidence [3,31]. Current cardio-oncology guidelines recommend opportunistic screening through pulse assessment or single-lead ECG at each clinical encounter [12]. However, more intensive strategies, such as serial ECG or ambulatory monitoring in higher-risk patients, remain insufficiently validated, and the role of wearable technologies is still evolving [67]. The occurrence of AF does not generally require discontinuation of BTKis therapy. Nonetheless, in patients at higher arrhythmic risk, the use of second-generation BTKis may be preferred when clinically feasible. Given the increased thromboembolic risk associated with AF, anticoagulation should be considered according to contemporary risk stratification models. However, in patients receiving BTKis, the decision to initiate anticoagulation must be carefully individualized, given the inherently increased bleeding risk associated with these agents. Thromboembolic risk assessment should be systematically performed using the CHA_2_DS_2_-VA score, in accordance with contemporary AF guidelines [68]. Patients with a CHA_2_DS_2_-VA score ≥1 should be considered for anticoagulation, although evidence specifically derived from BTKis-treated populations remains limited. In this context, direct oral anticoagulants are generally preferred over vitamin K antagonists. Among these, factor Xa inhibitors are typically favored due to their more predictable pharmacokinetic profile and lower potential for drug–drug interactions. In contrast, dabigatran should generally be avoided, as its dependence on P-glycoprotein transport may lead to increased drug exposure in the presence of BTKis, thereby amplifying bleeding risk [10,26]. Management of rate and rhythm control largely follows general AF guidelines, as dedicated evidence in BTKis-treated patients is lacking. Beta-blockers are typically preferred as first-line agents due to their safety profile. In contrast, caution is required with non-dihydropyridine calcium channel blockers and certain antiarrhythmic drugs, such as amiodarone, due to CYP3A4-mediated drug–drug interactions that may increase BTKis exposure. In addition, BTKis may increase digoxin levels through P-glycoprotein inhibition, with a potential risk of toxicity (Figure 2).

**Key points**
Most common cardiovascular toxicity, often underestimated without systematic monitoring; incidence increases over time and is higher with first-generation BTKis.Risk is driven by patient profile, particularly age, prior AF, and structural atrial disease; systematic screening (including single-lead ECG or wearable devices) may improve detection.Management favors a rate-control strategy and individualized anticoagulation, balancing thromboembolic and bleeding risk; beta-blockers are preferred, DOACs (particularly factor Xa inhibitors) are favored, and treatment modification is rarely required but may be considered in high-risk patients.

### 4.4. Ventricular Arrhythmias

VAs represent a less frequent but potentially severe cardiovascular complication associated with BTKis therapy. Emerging evidence indicates that BTKis exposure is associated not only with atrial arrhythmias but also with an increased risk of ventricular tachycardia (VT), ventricular fibrillation (VF), and sudden cardiac death (SCD) [10]. Early clinical analyses in patients with hematologic malignancies treated with ibrutinib reported an incidence of VA and/or SCD of approximately 6.0–7.9 per 1000 person-years, corresponding to a markedly higher risk compared with expected rates [69]. Consistent findings from clinical datasets suggest that approximately 1% of patients exposed to ibrutinib may experience SCD, underscoring the clinical relevance of this complication despite its relatively low absolute incidence [70]. Data on next-generation BTKis remain more limited but indicate that the risk is not fully abrogated. In cardiac-focused analyses of patients treated with acalabrutinib, an increased relative risk of ventricular arrhythmic events has been reported, although with a lower absolute incidence compared with ibrutinib [71]. Similarly, isolated cases of VAs have been described with zanubrutinib, supporting the concept of at least a partial class effect [72]. Taken together, these findings indicate a consistent, generation-dependent VAs signal that is best documented with ibrutinib, whereas evidence for newer agents remains comparatively sparse. The incidence and clinical evidence of BTKis-associated VAs are summarized in Table 5 [69,70,71,72,73]. Endpoints vary across studies, with some analyses reporting composite outcomes (including VAs, cardiac arrest, and SCD and others focusing on isolated VAs; incidence estimates should therefore be interpreted in this context. Identification of patients at increased risk remains challenging. Unlike AF, VAs do not appear to be consistently associated with pre-existing cardiovascular disease, although advanced age has been suggested as a relevant risk factor [72]. Emerging evidence points to a potential role of structural myocardial abnormalities. Cardiac magnetic resonance (CMR) studies have demonstrated a high prevalence of late gadolinium enhancement in patients treated with BTKis, suggesting myocardial fibrosis as a possible arrhythmogenic substrate [73]. Preclinical data further support a mechanistic basis, including impaired intracellular calcium handling and direct electrophysiological effects on ventricular cardiomyocytes, indicating a complex interplay between drug-specific effects and underlying myocardial vulnerability. From a clinical perspective, baseline cardiovascular assessment is essential. A baseline ECG should be obtained in all patients prior to initiation of BTKis therapy, with periodic ECG monitoring, particularly during the first year of treatment [7]. Baseline echocardiography should be considered in patients with cardiovascular risk factors or suspected structural heart disease. During follow-up, a low threshold for investigating symptoms suggestive of VAs—such as palpitations, syncope, dyspnea, or unexplained chest discomfort—is warranted [15]. Management requires close multidisciplinary collaboration. In patients with prior VAs, significantly reduced LVEF, or advanced HF [74], alternative oncologic strategies—such as time-limited regimens based on BCL-2 inhibition—should be considered when clinically appropriate. In cases of new-onset or symptomatic VAs during BTKis therapy, temporary interruption is generally warranted, together with prompt cardiologic evaluation, including echocardiography and, when indicated, CMR imaging. In patients with recurrent or sustained VT, treatment discontinuation may be required, depending on clinical severity and arrhythmic burden [7]. The potentially fatal events underscore the importance of early recognition and prompt access to defibrillation [75,76].

**Key points**
Rare but potentially life-threatening toxicity, including VT and SCD, more frequently observed with ibrutinib but not abolished with newer agents.Risk stratification remains challenging; advanced age and myocardial abnormalities (e.g., fibrosis on CMR) may identify vulnerable patients.Requires prompt evaluation and often impacts treatment strategy, including interruption, advanced cardiac imaging, and potential discontinuation in severe or recurrent cases.

### 4.5. Bleeding

Bleeding should be interpreted as a patient-level toxicity rather than a purely drug-related adverse event in patients with CLL treated with BTKis. Across the class, hemorrhagic events are among the most frequent non-cardiac toxicities, but their clinical expression is heterogeneous: low-grade bruising, petechiae, and epistaxis are common, whereas major bleeding is less frequent. This toxicity is best characterized with ibrutinib, for which the largest body of evidence is available [3,75,76]. In a prospective cohort of patients with CLL treated with single-agent ibrutinib, bleeding was reported in approximately 9% of cases [77]. In pooled analyses, any-grade bleeding occurred in up to 40% of patients, while major hemorrhage remained uncommon [55]. Similarly, meta-analyses of randomized trials confirmed an increased risk of both overall bleeding (32.8%) and major bleeding (3.0%) compared with comparator regimens [78]. Across cohort studies, pooled analyses, and randomized-trial meta-analyses, bleeding rates converge on a consistent pattern—frequent low-grade events and uncommon but non-negligible major hemorrhage—supporting a genuine, class-related signal with ibrutinib. Comparative data suggest a lower hemorrhagic burden with more selective BTKis, particularly acalabrutinib and zanubrutinib, although evidence for pirtobrutinib remains limited [79,80,81]. The incidence and clinical evidence of bleeding associated with BTKis therapy are summarized in Table 6 [21,22,36,77,78,79,81,82]. Mechanistically, BTKi-associated bleeding is thought to reflect off-target inhibition of Tec family kinases—particularly Tec itself—within platelets, which are essential for glycoprotein VI (collagen receptor), and von Willebrand factor–glycoprotein Ib-mediated signaling; this impairs collagen-induced platelet aggregation and adhesion independently of platelet count, and is more pronounced with less selective, first-generation agents [3]. Beyond frequency, the clinically relevant question is when bleeding occurs and in whom. Available studies suggest that first events often occur early after treatment initiation, although timing varies across cohorts, with median onset ranging from weeks to months [69,79,81]. In some series, the cumulative incidence appears to plateau after the first 6 months, suggesting that low-grade bleeding is particularly prominent early during treatment. However, major bleeding may occur throughout therapy, particularly in older and comorbid patients receiving concomitant antiplatelet or anticoagulant therapy. Real-world data highlight the central role of treatment interactions. Concurrent anticoagulation has emerged as a major determinant of hemorrhagic risk. A nationwide nested case–control study reported an approximately 2.5-fold increase in clinically relevant bleeding among patients receiving BTKis together with anticoagulants [82], while a population-based cohort showed a 2.7-fold higher hazard of bleeding with concomitant ibrutinib and oral anticoagulation, with a stronger signal in warfarin-exposed patients [83]. Bleeding assessment remains primarily clinical and individualized. In routine practice, evaluation should focus on bleeding severity and pattern, concomitant antithrombotic therapy, platelet count, comorbidity burden, and recent invasive procedures. Although platelet dysfunction and alterations in von Willebrand factor or factor VIII may contribute to inter-individual variability, these parameters are not routinely used in clinical decision-making [84]. Management should be tailored to the overall therapeutic context. Concomitant BTKis and antiplatelet or anticoagulant therapy requires particular caution, with preference generally given to direct oral anticoagulants over vitamin K antagonists when anticoagulation is indicated. Potentially avoidable contributors to bleeding should be systematically reassessed, particularly aspirin used for primary prevention and unnecessary combinations of antithrombotic agents. When dual antiplatelet therapy is required, BTKis are generally not preferred, and cardiology input is advisable. In patients requiring long-term anticoagulation or experiencing recurrent bleeding on ibrutinib, switching to a more selective BTKis—or, when clinically appropriate, to a fixed-duration non-BTK-based regimen—may be more appropriate than repeated dose interruptions [7,15].

**Key points**
Common but predominantly low-grade toxicity, with early onset and a heterogeneous clinical presentation; major bleeding is less frequent but clinically relevant.Management is centered on minimizing modifiable bleeding risk (especially antithrombotic burden) rather than stopping therapy; treatment switch to more selective BTKis or alternative regimens should be considered in patients with persistent high-risk profiles or recurrent events.

### 4.6. Drug Interactions Between BTK Inhibitors and Cardiology Drugs

Drug–drug interactions between BTKis and cardiovascular therapies are a key determinant of treatment tolerability in CLL patients, who are typically older, multimorbid, and exposed to polypharmacy. Three mechanisms are clinically relevant: CYP3A-mediated interactions, transporter-mediated interactions, and pharmacodynamic amplification of bleeding risk (Table 7). Covalent BTKis are CYP3A substrates, and concomitant use of CYP3A inhibitors may increase drug exposure and toxicity. This is particularly relevant in cardio-oncology, where verapamil, diltiazem, and amiodarone may increase BTKis levels and should be avoided when feasible or used with caution. Accordingly, in patients who develop AF during BTKis therapy, beta-blockers are preferred for rate control, whereas non-dihydropyridine calcium-channel blockers should be used cautiously [84,85]. Ibrutinib is the most interaction-prone agent, due to both CYP3A metabolism and P-glycoprotein interactions. Digoxin and dabigatran require particular caution, with dose separation and closer monitoring when co-administered [84,85]. Anticoagulation represents a major challenge. Vitamin K antagonists are generally avoided, while direct oral anticoagulants (DOACs) are preferred when indicated. However, their use must be individualized, as interaction burden is both pharmacokinetic and pharmacodynamic, particularly in patients with high bleeding risk or concomitant antiplatelet therapy [80,84,85]. Second-generation BTKis partly reduce, but do not eliminate, the interaction burden. Although acalabrutinib and zanubrutinib remain CYP3A substrates, their improved selectivity may facilitate treatment continuation in patients requiring complex cardiovascular therapy. Pirtobrutinib shows a favorable profile, but careful co-medication review remains necessary [82,86]. The most clinically relevant interactions are pharmacodynamic. BTK inhibition impairs platelet function, and combination with antiplatelet agents or anticoagulants may significantly increase bleeding risk, particularly in patients with AF or coronary artery disease. Accordingly, medication reconciliation should be mandatory at treatment initiation and at each clinical event. Aspirin for primary prevention should generally be discontinued, and dual or triple antithrombotic therapy should prompt multidisciplinary reassessment. In patients requiring long-term interacting therapies, switching from ibrutinib to a more selective BTKi may be preferable to repeated treatment interruptions [80,83,84].

**Key points**
CYP3A-mediated interactions are the main pharmacokinetic driver and should guide cardiovascular drug selection.Ibrutinib has the highest interaction burden; second-generation BTKis may improve tolerability in complex patients.Bleeding risk is primarily pharmacodynamic and driven by concomitant antithrombotic therapy.

## 5. A Practical Framework for Cardiovascular Management in Patients Receiving BTK Inhibitors

**Figure 3:**
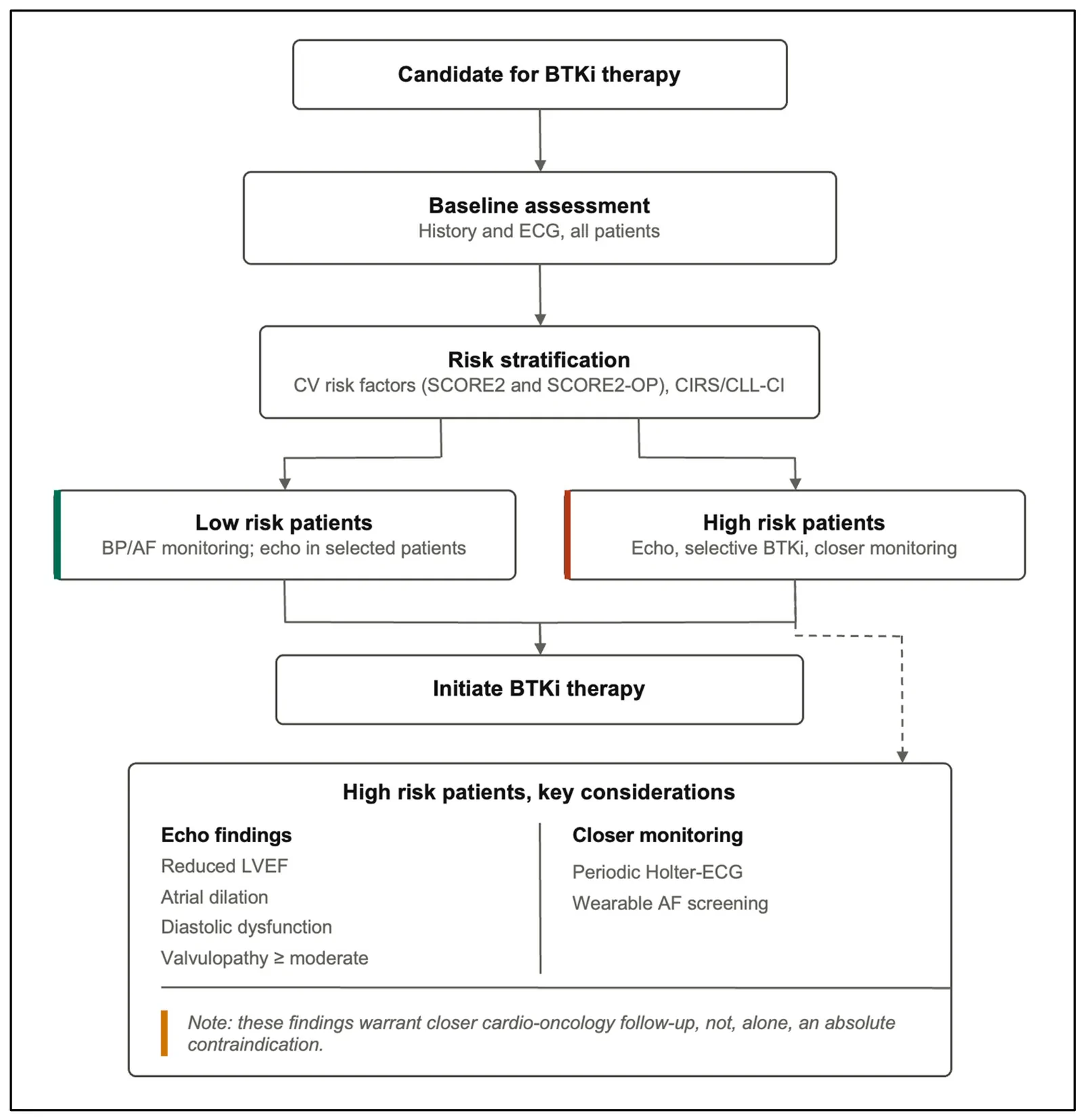
Baseline cardiovascular risk stratification and monitoring algorithm before initiation of BTK inhibitor therapy. Abbreviations: AF, atrial fibrillation; BP, blood pressure; BTKi, Bruton tyrosine kinase inhibitor; CIRS, Cumulative Illness Rating Scale; CLL-CI, CLL-specific Comorbidity Index; CV, cardiovascular; ECG, electrocardiogram; Echo, echocardiography; LVEF, left ventricular ejection fraction.

The cardiovascular management of patients receiving BTKis should follow a structured, patient-centered approach integrating baseline assessment, risk stratification, longitudinal monitoring, and event-driven treatment adaptation (Figure 3). At treatment initiation, careful evaluation of cardiovascular history, comorbidities, ECG, echocardiographic profile when indicated, co-medications, and antithrombotic burden is essential. Routine measurement of NT-proBNP prior to BTKi initiation is not currently supported by specific guidance or dedicated evidence, but its use appears reasonable in patients with symptoms suggestive of de novo HF or with a prior history of HF. During follow-up, blood pressure monitoring and opportunistic AF screening should be systematically implemented, while vigilance for HF, VAs, and bleeding should remain high, particularly in vulnerable patients and during prolonged exposure. When toxicity occurs, management should not focus on the event alone, but on its interaction with patient profile, competing risks, and the need to preserve treatment continuity. Within this framework, dose modification, temporary interruption, switching to a more selective BTKi, or transition to alternative time-limited strategies should be tailored through close multidisciplinary collaboration between hematologists and cardiologists, balancing optimal disease control with the prevention and management of cardiovascular complications. Key practical recommendations across all six toxicities are summarized in Table 8.

## 6. Conclusions

BTKis have transformed the management of CLL and other B-cell malignancies, but their clinical use is increasingly shaped by cardiovascular safety. In contemporary practice, cardiovascular toxicity should not be interpreted as an isolated drug-related event, but as a dynamic determinant of treatment continuity, competing risk, and overall outcome in an older, multimorbid population. Among these toxicities, AF and HTN are the most frequent and clinically visible, whereas HF and VAs, although less common, may have substantial prognostic and therapeutic implications. Bleeding risk further complicates management, particularly in patients requiring concomitant antithrombotic therapy. Taken together, these complications highlight that the management of patients receiving BTKis cannot rely on a toxicity-specific approach alone. Rather, it requires a patient-centered framework integrating baseline cardiovascular risk, comorbidity burden, co-medications, treatment duration, and the expected need for long-term oncologic disease control. Unlike existing guidance, including the ESC cardio-oncology guidelines [12] and international consensus statements on BTKis cardiovascular management [86], which address each cardiovascular complication largely in isolation, the framework proposed here explicitly anchors clinical decision-making in patient-level comorbidity burden and vulnerability (Section 3) and translates this into toxicity-specific, actionable management algorithms (Figure 1, Figure 2 and Figure 3) spanning the full spectrum of BTKi-associated cardiovascular complications. In this context, the choice of BTKis is itself part of cardiovascular decision-making, as different agents are associated with distinct toxicity profiles and different degrees of pharmacologic complexity. A structured cardio-oncology approach based on baseline assessment, early recognition of toxicity, individualized monitoring, and multidisciplinary treatment adjustment is therefore essential. The goal is not only to reduce cardiovascular events, but also to preserve the feasibility and continuity of effective anticancer therapy. As the use of BTKis continues to expand, optimizing this balance will become an increasingly important component of precision care in CLL.

## 7. Future Directions

Several unmet needs remain in the cardiovascular management of patients treated with BTKis. First, prospective studies with systematic cardiovascular surveillance are needed to better define the true incidence, timing, and clinical trajectory of AF, HTN, HF, and VAs across different BTKis. Current evidence is limited by heterogeneous definitions, variable follow-up, and inconsistent monitoring strategies, which likely lead to under-recognition of clinically relevant events. Second, risk stratification requires refinement. Clinical variables such as age, prior cardiovascular disease, baseline HTN, prior AF, and reduced LVEF are clearly relevant, but they are unlikely to capture the full spectrum of vulnerability. Future research should evaluate integrated models combining clinical profile, structural cardiac markers, biomarkers, and, where appropriate, ambulatory or wearable-based monitoring strategies in order to identify patients most likely to benefit from intensified surveillance or alternative therapeutic choices. Third, more data are needed on the comparative cardiovascular safety of newer-generation and non-covalent BTKis in real-world populations, particularly in older and highly comorbid patients who are underrepresented in clinical trials. This is especially important for understanding whether improved kinase selectivity translates into a clinically meaningful reduction in long-term cardiovascular burden and drug–drug interaction complexity. Fourth, beyond currently approved agents, a new class of BTK-targeting therapies—covalent-independent BTK degraders such as bexobrutideg (NX-2127), NX-5948, and BGB-16673—is entering clinical development, with early efficacy demonstrated even in patients resistant to covalent and non-covalent BTKis [87]. Their cardiovascular safety profile remains entirely uncharacterized, representing an immediate priority for future cardio-oncology surveillance as these agents progress through clinical trials. Finally, management strategies themselves need stronger evidence, as most current recommendations rely on observational data, retrospective cohorts, or expert consensus rather than dedicated randomized trials. Key open questions include the optimal monitoring schedule, the most appropriate anticoagulation strategy in BTKis-related AF, the role of preventive cardiovascular therapies, the thresholds for treatment interruption, switching, or permanent discontinuation, and the potential role of rechallenge strategies after severe cardiotoxicity, as explored in other settings such as immune checkpoint inhibitors [88]. Future progress will likely depend on closer integration between hematology and cardio-oncology, as well as on the development of pragmatic care pathways that can be applied in routine clinical practice.

## Figures and Tables

**Table 1:** Main characteristics, clinical use, and cardiovascular profile of available BTK inhibitors.

Drug	Generation	Binding Type	Selectivity (Relative)	Half-Life	Primary Clearance Route	Clinical Use in CLL	Cardiovascular Profile (Key Features)	Key Trials/Evidence
Ibrutinib	1st	Covalent, irreversible (BTK Cys481)	Low (off-target activity)	4–6 h	Hepatic (CYP3A4); predominantly fecal excretion (~80%), minimal renal (<10%)	Frontline and R/R; continuous therapy	Highest risk of AF; cumulative HTN; increased bleeding risk; rare HF and VAs	RESONATE [2]; E1912 [18]
Acalabrutinib	2nd	Covalent, irreversible	Improved vs. ibrutinib	~1 h (parent); ~7 h (active metabolite ACP-5862)	Hepatic (CYP3A4); fecal 84%, renal 12%	Frontline and R/R (±anti-CD20)	Lower AF vs. ibrutinib; reduced HTN burden; residual cardiovascular risk persists	ELEVATE-TN [19]; ASCEND [20]; ELEVATE-RR [21]
Zanubrutinib	2nd	Covalent, irreversible	High selectivity	~2–4 h	Hepatic (CYP3A4); predominantly fecal excretion (~87%), minimal renal (<1%)	Frontline and R/R; expanding role	Reduced AF incidence; HTN remains clinically relevant; overall improved tolerability	ALPINE [22]; SEQUOIA [23]
Pirtobrutinib	Non-covalent (3rd gen)	Reversible	Highly selective; active vs. C481	~19–27 h	Hepatic (CYP3A substrate)	R/R after prior BTKis	Low incidence of AF and bleeding (early data); long-term cardiovascular profile not fully characterized	BRUIN [24]; ongoing phase III

Abbreviations: AF, atrial fibrillation; CYP3A4, cytochrome P450 3A4; HF, heart failure; HTN, arterial hypertension; VAs, ventricular arrhythmias; BTK, Bruton tyrosine kinase; BTKis, Bruton tyrosine kinase inhibitors; R/R, relapsed/refractory; CLL, chronic lymphocytic leukemia; anti-CD20, anti-CD20 monoclonal antibody; PFS, progression-free survival.

**Table 2:** Incidence and characteristics of hypertension associated with BTK inhibitors.

Study (Author, Year)	Study Design	BTKi Evaluated	Hypertension Incidence	Key Findings
Brown et al., 2021 (ELEVATE-RR) [21]	Phase III RCT	Acalabrutinib vs. Ibrutinib	9.4% vs. 23.2% (any grade)	Improved cardiovascular safety profile
Burger et al., 2015 [30]	Phase III RCT (RESONATE)	Ibrutinib	~14% (any grade); ~4% (grade ≥ 3)	Early trials likely underestimated incidence
Byrd et al., 2020 [31]	Phase Ib/II (long-term follow-up)	Ibrutinib	NR	HTN incidence increases with treatment duration
Kittai et al., 2024 [32]	Matching-adjusted indirect comparison	Acalabrutinib vs. Zanubrutinib	OR < 1 (acalabrutinib vs. zanubrutinib)	Suggests differential vascular effects among 2nd-gen BTKis
Chen et al., 2022 [33]	Observational study	Next-generation BTKis	OR 0.44 (acalabrutinib monotherapy vs. zanubrutinib); OR 0.52 (acalabrutinib + obinutuzumab vs. zanubrutinib)	Association with CV events including HTN
Samples et al., 2024 [34]	Multicenter retrospective study	Ibrutinib (±others)	48.9% (new/worsened HTN); 53.9% at 1 year (SBP ≥ 130 mmHg)	Combination antihypertensive therapy required
Dickerson et al., 2019 [35]	Observational cohort	Ibrutinib	Up to ~78% (new/worsening HTN)	High real-world incidence; progressive over time
Tam et al., 2020 (ASPEN) [36]	Phase III RCT	Zanubrutinib vs. Ibrutinib	14.9% vs. 25.5%	Lower incidence with second-generation BTKis

Abbreviations: BTK, Bruton tyrosine kinase; BTKis, Bruton tyrosine kinase inhibitors; HTN, arterial hypertension; NR, not reported; OR, odds ratio; RCT, randomized controlled trial.

**Table 3:** Incidence and clinical evidence of heart failure with BTK inhibitors.

Study (Author, Year)	Study Design	BTKi Evaluated	HF Incidence	Key Findings
Munir et al., 2019 [40]	Long-term follow-up (6 years)	Ibrutinib	Not systematically reported	Cardiovascular toxicity, including HF, emerges with prolonged exposure
Abdel-Qadir et al., 2021 [41]	Population-based cohort	Ibrutinib	7.7% vs. 3.6% (control)	Significantly higher HF risk compared with chemotherapy-treated patients
Salem et al., 2019 [42]	Pharmacovigilance analysis	Ibrutinib	ROR > 3	Strong safety signal for HF with ibrutinib in real-world data
Brown et al., 2022 [43]	Pooled analysis (*n* = 762)	Acalabrutinib	<1%	Low HF incidence with second-generation BTKis, suggesting improved cardiovascular safety

Abbreviations: BTK, Bruton tyrosine kinase; BTKis, Bruton tyrosine kinase inhibitors; HF, heart failure; ROR, Reporting Odds Ratio.

**Table 4:** Incidence and clinical evidence of atrial fibrillation with BTK inhibitors.

Study (Author, Year)	Study Design	BTKi Evaluated	AF Incidence	Key Findings
Byrd et al., 2014 [2]	Phase III randomized controlled trial	Ibrutinib	10/195 (5.1%)	AF incidence higher in the Ibrutinib group compared with the ofatumumab group
Tam et al., 2020 [3]	Phase III randomized controlled trial	Ibrutinib	15/98 (15.3%)	Higher incidence of AF in the Ibrutinib group compared with Zanubrutinib (2/101 (2.0%))
Sharman et al., 2020 [19]	Phase III randomized controlled trial	Acalabrutinib	7/179 (3.9%)	Acalabrutinib with Obinutuzumab or not improved outcome, but higher incidence rate of AF (7/179 vs. 1/169)
Ghia et al., 2022 [20]	Phase III randomized controlled trial	Acalabrutinib	3/154 (1.9%)	Low incidence of AF and lower other adverse events with Acalabrutinib compared with chemoimmunotherapy
Mato et al., 2023 [24]	Phase I-II trial	Pirtobrutinib	12/317 (3.8%)	Low incidence of AF reported with a median follow up of 19.4 months
Burger et al., 2015 [30]	Phase III randomized controlled trial	Ibrutinib	8/136 (5.9%)	AF incidence higher in the Ibrutinib group compared with Chlorambucil, but with lower occurrence of adverse events
Byrd et al., 2021 [31]	Phase III randomized controlled trial	Ibrutinib	41/263 (15.6%)	AF 2.4 times higher in the Ibrutinib group with shorter median onset time compared with Acalabrutinib
Woyach et al., 2018 [50]	Phase III randomized controlled trial	Ibrutinib-based regimens	6–16%	AF identified as a clinically relevant toxicity in older patients treated with ibrutinib
Wiczer et al., 2017 [51]	Retrospective cohort	Ibrutinib	Median onset ~5–8 months	Defined timing, cumulative incidence, and clinical management of AF
Alexandre et al., 2023 [52]	Meta-analysis (phase II–III trials)	Multiple anticancer agents (including BTKis)	4.92 per 100 person-years (ibrutinib)	Highest AF incidence observed with ibrutinib among anticancer monotherapies
Baptiste et al., 2019 [53]	Prospective cardiac surveillance study	Ibrutinib	Up to 38% at 24 months	Systematic ECG monitoring revealed substantial underestimation of AF in clinical trials
Ojo et al., 2023 [54]	Real-world cohort study	BTKis (mainly ibrutinib)	25% at 5 years	BTKi exposure associated with markedly increased long-term AF risk
Brown et al., 2017 [55]	Pooled analysis of randomized trials	Ibrutinib	Not uniformly reported	Advanced age and prior AF identified as major predictors of AF events
Wang et al., 2016 [56]	Phase II randomized controlled trial	Ibrutinib	7/50 (14.0%)	AF associated with age and cardiac toxicity
Dimopoulos et al., 2018 [57]	Phase III randomized controlled trial	Ibrutinib	11/75 (14.7%)	Higher incidence of AF in the Ibrutinib-Rituximab group compared to placebo-rituximab group
Wang et al., 2018 [58]	Phase II single arm trial	Acalabrutinib	0/124 (0.0%)	AF not detected in patients treated with Acalabrutinib with a median follow up of 15.2 months
Xu et al., 2020 [59]	Phase II trial	Zanubrutinib	0/91 (0.0%)	AF not detected in patients treated with Zanubrutinib with a median follow up of 15.1 months
Opat et al., 2021 [60]	Phase II trial	Zanubrutinib	2/68 (2.9%)	Low incidence of AF reported with a median follow up of 15.7 months
Woyach et al., 2025 [61]	Phase III randomized controlled trial	Pirtobrutinib	8/330 (2.4%)	Lower incidence of AF reported compared with Ibrutinib (44/325 (13.5%))

Abbreviations: AF, atrial fibrillation; BTK, Bruton tyrosine kinase; BTKis, Bruton tyrosine kinase inhibitors.

**Table 5:** Incidence and clinical evidence of ventricular arrhythmias with BTK inhibitors.

Study (Author, Year)	Study Design	BTKi Evaluated	Endpoint Definition	VAs Incidence (%)	Key Findings
Lampson et al., 2017 [69]	Retrospective analysis (clinical trials + FAERS)	Ibrutinib	Composite: VAs, cardiac arrest, SCD	~1%	Increased rates vs. expected population; signal supported by randomized data (HELIOS)
Guha et al., 2018 [70]	Retrospective cohort	Ibrutinib	Incident symptomatic VAs (VT/VF/PVCs)	1.9%	Markedly increased risk vs. general population (RR ~12), independent of structural heart disease
Bhat et al., 2022 [71]	Retrospective cohort	Acalabrutinib	Incident symptomatic VAs (VT/VF/PVCs)	~2.8%	Residual risk persists with second-generation BTKis (RR ~8), suggesting partial class effect
Fazal et al., 2023 [72]	Case series/literature-based cohort	Multiple TKIs (including BTKis)	Clinically reported VAs	Not systematically quantified	Heterogeneous presentations; supports a possible class effect
Buck et al., 2023 [73]	CMR-based observational study	Ibrutinib	Structural substrate (fibrosis; LGE)	Not applicable	High prevalence of LGE, suggesting an arrhythmogenic substrate

Footnotes: Endpoints vary across studies (composite vs. isolated VAs), and incidence estimates should be interpreted accordingly. Abbreviations: PVCs, premature ventricular contractions; SCD, sudden cardiac death; VAs, ventricular arrhythmias; VT, ventricular tachycardia; VF, ventricular fibrillation.

**Table 6:** Incidence and clinical evidence of bleeding with BTK inhibitors.

Study (Author, Year)	Study Design	BTKi Evaluated	Endpoint Definition	Bleeding Incidence (%)	Key Findings
Seymour et al., 2023 (ELEVATE-RR) [21]	Phase III RCT (post hoc safety analysis)	Acalabrutinib vs. Ibrutinib	Any-grade bleeding (AE of clinical interest)	Higher with ibrutinib (no % in primary report)	Consistently higher bleeding burden with ibrutinib
Brown et al., 2019 [22]	Pooled analysis (15 clinical trials)	Ibrutinib	Any-grade bleeding; major bleeding	~40% (any grade); major bleeding uncommon	High overall incidence; mostly low-grade events
Tam et al., 2020 (ASPEN) [36]	Phase III RCT	Zanubrutinib vs. Ibrutinib	Hemorrhagic events (any grade; major bleeding)	~4% (major bleeding with zanubrutinib)	Lower bleeding incidence vs. ibrutinib
Mato et al., 2018 [77]	Multicenter retrospective cohort	Ibrutinib	Clinically reported bleeding events (any grade)	~9%	Bleeding contributed to treatment discontinuation
Wang et al., 2020 [78]	Systematic review and meta-analysis of RCTs	Ibrutinib vs. comparators	Overall bleeding (any grade); major bleeding (CTCAE ≥ 3 or serious)	RR 2.56 (overall); RR 2.08 (major)	Significantly increased bleeding risk vs. control regimens
Brown et al., 2024 [79]	Pooled safety database	Zanubrutinib vs. Ibrutinib	Treatment-emergent hemorrhage	Lower exposure-adjusted incidence with zanubrutinib	Improved safety vs. ibrutinib over time
Lipsky et al., 2015 [81]	Prospective cohort	Ibrutinib	Bleeding-related AEs (grade ≤ 2; CTCAE-based)	55% (grade ≤ 2); ~5–6% grade ≥ 3 in the literature	Early onset; platelet dysfunction-mediated
Allouchery et al., 2023 [82]	Population-based nested case–control	Ibrutinib	Clinically relevant bleeding (with anticoagulation)	~2.5-fold increased risk	Strong interaction with antithrombotic therapy

**Footnotes:** 1. Bleeding definitions varied (any-grade vs. major; CTCAE-based). 2. Major bleeding generally defined as CTCAE grade ≥3 or serious events. 3. Exposure-adjusted and pooled data are not directly comparable across studies. 4. Real-world studies often report relative risk rather than incidence. Abbreviations: AE, adverse event; BTKis, Bruton tyrosine kinase inhibitors; CTCAE, Common Terminology Criteria for Adverse Events; RCT, randomized controlled trial; RR, risk ratio; TEAE, treatment-emergent adverse events.

**Table 7:** Drug–drug interactions between BTK inhibitors and cardiovascular therapies: mechanisms and associated risks.

Mechanism	Cardiovascular Drugs Involved	Pharmacological Consequence	Clinical Risk
CYP3A-mediated interactions	Verapamil, diltiazem, amiodarone	CYP3A inhibition → increased BTKi plasma concentration	Increased risk of AF, HTN, bleeding, and overall toxicity
Transporter-mediated interactions (P-glycoprotein)	Digoxin	Reduced efflux → increased digoxin levels	Risk of digoxin toxicity (arrhythmias, conduction disturbances)
Transporter-mediated interactions (P-glycoprotein)	Dabigatran	Increased dabigatran exposure	Increased bleeding risk
Pharmacodynamic amplification of bleeding risk	Aspirin, P2Y12 inhibitors	Additive platelet dysfunction	Increased bleeding risk
Pharmacodynamic amplification of bleeding risk	DOACs/VKAs	Combined anticoagulant effect + impaired platelet function	Increased major bleeding risk

Abbreviations: BTKi, Bruton tyrosine kinase inhibitor; CYP3A, cytochrome P450 3A; AF, atrial fibrillation; HTN, hypertension; DOACs, direct oral anticoagulants; VKAs, vitamin K antagonists.

**Table 8:** Clinical Pearls for Everyday Practice.

Domain	Clinical Pearl
Baseline evaluation	CV history and risk factor assessment and correction; ECG in all patients; echocardiography in high-risk patients (CV risk factors or suspected structural heart disease).
Monitoring	Home BP monitoring; opportunistic AF screening at each visit; periodic ECG in the first year if VA risk.
HTN management	DHP-CCBs/RAS inhibitors first-line; avoid non-DHP CCBs (CYP3A4 interaction). Combination often needed: β-blocker + thiazide in pre-existing HTN, ACE-i/ARB + thiazide in de novo HTN. Close monitoring and prompt intensification for rising BP; discontinuation rarely needed—reserve dose modification/interruption/switch for severe, uncontrolled, or resistant HTN.
AF management	Rate control favored (β-blockers first-line); avoid non-DHP CCBs/amiodarone (CYP3A4 interaction). CHA2DS2-VA–guided anticoagulation, individualized against bleeding risk; DOACs (factor Xa inhibitors) preferred over VKAs; avoid dabigatran (P-glycoprotein interaction).
Interruption/switching	Rarely needed for HTN/AF; consider for HF grade ≥3, recurrent/sustained VT, or recurrent bleeding; second-generation BTKi preferred in high-risk patients.

Abbreviations: ACE, angiotensin-converting enzyme; AF, atrial fibrillation; ARB, angiotensin receptor blocker; BP, blood pressure; BTKi, Bruton tyrosine kinase inhibitor; CCB, calcium channel blocker; CHA_2_DS_2_-VA, stroke risk score used for anticoagulation decision-making in atrial fibrillation; CV, cardiovascular; CYP3A4, cytochrome P450 3A4; DHP, dihydropyridine; DOAC, direct oral anticoagulant; ECG, electrocardiogram; HF, heart failure; HTN, arterial hypertension; RAS, renin–angiotensin system; VA, ventricular arrhythmia; VKA, vitamin K antagonist; VT, ventricular tachycardia.

## Data Availability

No new data were created or analyzed in this study. Data sharing is not applicable to this article.

## Abbreviations

The following abbreviations are used in this manuscript:

ACE	Angiotensin-Converting Enzyme
AF	Atrial Fibrillation
ARB	Angiotensin Receptor Blocker
BCL-2	B-cell Lymphoma 2
BP	Blood Pressure
BTK	Bruton Tyrosine Kinase
BTKi/BTKis	Bruton Tyrosine Kinase Inhibitors
CIRS	Cumulative Illness Rating Scale
CLL	Chronic Lymphocytic Leukemia
CLL-CI	CLL-specific Comorbidity Index
CMR	Cardiac Magnetic Resonance
CTCAE	Common Terminology Criteria for Adverse Events
CV	Cardiovascular
CYP3A	Cytochrome P450 3A
DOAC	Direct Oral Anticoagulant
ECG	Electrocardiogram
Echo	Echocardiography
ESC	European Society of Cardiology
FAERS	FDA Adverse Event Reporting System
FDA	Food and Drug Administration
GDMT	Guideline-Directed Medical Therapy
HF	Heart Failure
HFrEF	Heart Failure with Reduced Ejection Fraction
HTN	Arterial Hypertension
IFN-γ	Interferon-Gamma
LGE	Late Gadolinium Enhancement
LVEF	Left Ventricular Ejection Fraction
MRA	Mineralocorticoid Receptor Antagonist
PI3K	Phosphoinositide 3-Kinase
PVC	Premature Ventricular Contraction
ROR	Reporting Odds Ratio
R/R	Relapsed/Refractory
SCD	Sudden Cardiac Death
SGLT2	Sodium-Glucose Cotransporter 2
TCR	T-Cell Receptor
TNF-α	Tumor Necrosis Factor-alpha
VA/VAs	Ventricular Arrhythmia/s
VF	Ventricular Fibrillation
VKA	Vitamin K Antagonist
VT	Ventricular Tachycardia
